# Complexation of fungal extracellular nucleic acids by host LL-37 peptide shapes neutrophil response to *Candida albicans* biofilm

**DOI:** 10.3389/fimmu.2024.1295168

**Published:** 2024-02-06

**Authors:** Magdalena Juszczak, Marcin Zawrotniak, Maria Rapala-Kozik

**Affiliations:** ^1^ Department of Comparative Biochemistry and Bioanalytics, Faculty of Biochemistry, Biophysics and Biotechnology, Jagiellonian University, Krakow, Poland; ^2^ Doctoral School of Exact and Natural Sciences, Jagiellonian University, Krakow, Poland

**Keywords:** neutrophils, NETs, *Candida albicans*, biofilm, LL-37

## Abstract

*Candida albicans* remains the predominant cause of fungal infections, where adhered microbial cells form biofilms - densely packed communities. The central feature of *C. albicans* biofilms is the production of an extracellular matrix (ECM) consisting of polymers and extracellular nucleic acids (eDNA, eRNA), which significantly impedes the infiltration of host cells. Neutrophils, as crucial players in the innate host defense, employ several mechanisms to eradicate the fungal infection, including NETosis, endocytosis, or the release of granules containing, among others, antimicrobial peptides (AMPs). The main representative of these is the positively charged peptide LL-37 formed from an inactive precursor (hCAP18). In addition to its antimicrobial functions, this peptide possesses a propensity to interact with negatively charged molecules, including nucleic acids. Our *in vitro* studies have demonstrated that LL-37 contacting with *C. albicans* nucleic acids, isolated from biofilm, are complexed by the peptide and its shorter derivatives, as confirmed by electrophoretic mobility shift assays. We indicated that the generation of the complexes induces discernible alterations in the neutrophil response to fungal nucleic acids compared to the effects of unconjugated molecules. Our analyses involving fluorescence microscopy, flow cytometry, and Western blotting revealed that stimulation of neutrophils with DNA:LL-37 or RNA:LL-37 complexes hamper the activation of pro-apoptotic caspases 3 and 7 and fosters increased activation of anti-apoptotic pathways mediated by the Mcl-1 protein. Furthermore, the formation of complexes elicits a dual effect on neutrophil immune response. Firstly, they facilitate increased nucleic acid uptake, as evidenced by microscopic observations, and enhance the pro-inflammatory response, promoting IL-8 production. Secondly, the complexes detection suppresses the production of reactive oxygen species and attenuates NETosis activation. In conclusion, these findings may imply that the neutrophil immune response shifts toward mobilizing the immune system as a whole, rather than inactivating the pathogen locally. Our findings shed new light on the intricate interplay between the constituents of the *C. albicans* biofilm and the host’s immune response and indicate possible reasons for the elimination of NETosis from the arsenal of the neutrophil response during contact with the fungal biofilm.

## 1 Introduction

*Candida albicans* has emerged as one of the most prevalent opportunistic human pathogens worldwide. Its clinical significance lies in the ability to transition from a harmless commensal member of the human microbiota to a formidable pathogen capable of causing a diverse array of infections, particularly in immunocompromised individuals. Candidiasis, the collective term for infections caused by *C. albicans*, encompasses a wide spectrum of clinical manifestations, ranging from superficial mucosal infections to life-threatening systemic candidemia and invasive candidiasis (1, 2).

*C. albicans* has evolved several mechanisms to establish persistent and recurrent infections. These strategies involve the utilization of a wide range of adhesins, which facilitate attachment to both biotic and abiotic surfaces. *Candida* exhibits phenotypic switching between different forms, including yeast, pseudohyphae, and hyphae, providing it with remarkable adaptability to avoid host immune responses and invade tissues. Furthermore, *C. albicans* secretes an array of extracellular enzymes, such as aspartic proteases (SAPs) and lipases, with broad-spectrum activity. In the case of SAPs, we distinguish 10 distinct proteins (SAP1–10), which degrade host proteins such as albumin, hemoglobin, keratin, collagen, laminin, fibronectin, mucin, as well as immunoglobulins and antimicrobial peptides (3–6). Additionally, it has recently been shown that *C. albicans* produces a toxin, called candidalysin (7). Among the various virulence factors exhibited by this yeast, the ability to form biofilms stands out as a crucial mechanism that enhances its resistance to host defenses and antifungal treatments, posing a significant challenge in clinical settings. Biofilms are complex microbial communities held together by an extracellular matrix (ECM) that offers structural support to the embedded microorganisms, leading to the recalcitrance of biofilm-associated infections (8, 9). The biofilm ECM is composed of polysaccharides, proteins, lipids, and extracellular nucleic acids (eDNA, eRNA), and its constituents vary among different species and environmental conditions enabling the biofilm to function as a cohesive, multicellular community (10–13). The phenomenon of eDNA and eRNA occurrence in biofilms has been well-known in bacteria for many years but in the case of *C. albicans*, it was first demonstrated in 2010 by Martis et al. (11, 14–16). Previous work indicated that extracellular nucleic acids, due to their physicochemical properties turned out to be an important factor contributing to fungal biofilm stability, adhesion, and resistance to antifungal agents (11, 17). However, the mechanism responsible for the release of extracellular nucleic acids by *C. albicans* during biofilm formation remains poorly understood. Some studies have indicated a potential link to chitinases’ activity, which is involved in cell wall remodeling during the formation of the hyphal form of the cells (17). Furthermore, yeast cells also transport various macromolecules, including nucleic acids, via extracellular vesicles (EVs) (18).

The development of a fungal infection drives the recruitment of immune cells. Neutrophils, as the first responders of the innate immune system, possess several effector mechanisms to combat fungal pathogens (19, 20). These strategies include phagocytosis, degranulation, and the formation of neutrophil extracellular traps (NETs) through a process called NETosis (21). NETs are web-like structures composed primarily of decondensed chromatin and various antimicrobial proteins. NETosis is activated by many fungal agents, mainly β-glucans, aspartyl proteases, quorum-sensing molecules, as well as extracellular nucleic acids (12, 22–24). This process is thought to be primarily dependent on the generation of reactive oxygen species (ROS) as an essential trigger. The pathway involves the activation of NADPH oxidase by protein kinase C (PKC) (25). It has also been demonstrated that neutrophils can release NETs without the activation of the NADPH oxidase in the ROS-independent pathway in response to some factors (25, 26). However, while NETosis acts as a powerful defense mechanism against microbial invaders, its effectiveness against mature *C. albicans* biofilms remains debatable (27, 28). Activation of NETosis or degranulation is associated with the release of significant amounts of cationic antimicrobial peptides (AMPs). Among the AMPs, the LL-37 peptide has garnered significant attention due to its multifunctional role in innate immunity. LL-37 is the C-terminal part of the only cathelicidin in humans – hCAP18, which is mainly expressed by neutrophils, epithelial cells, and keratinocytes (29, 30). LL-37 possesses a diverse range of functions beyond its direct antimicrobial activity. It exerts effects on various processes, including chemotaxis, activation of epithelial cells, angiogenesis, epithelial wound repair, and induction of chemokine secretion (31). Furthermore, the tertiary structure of LL-37, coupled with its net positive charge and amphipathic properties, facilitates its interaction with negatively charged molecules such as bacterial lipids, lipoteichoic acid (LTA), lipopolysaccharide (LPS), and nucleic acids (32–35). These interactions can significantly impact the dynamic interplay between the host and pathogens during infection. In the case of LPS and LTA molecules, it has been shown that the presence of LL-37 inhibits LPS/LTA-induced pro-inflammatory response (36). Research on DNA/RNA:LL-37 complexes has been conducted mainly in the context of the autoimmune response, where it has been repeatedly shown that LL-37 converts self-DNA and self-RNA into a potent trigger of inflammation. It has been demonstrated that the formation of complexes between self-DNA and the LL-37 peptide leads to increased expression of type I interferons in plasmacytoid dendritic cells in the course of type I diabetes, psoriasis and systemic lupus erythematosus (37–39). Elevated amounts of the LL-37-mitochondrial DNA complex are also observed in the plasma and plaques of patients suffering from atherosclerosis (40).

The effect of the LL-37 peptide on the modulation of the neutrophil response to *C. albicans* infection has not been studied so far. In our current study, we have shown for the first time that the interactions between *C. albicans* nucleic acids and the LL-37 peptide lead to significant changes in the neutrophil immune response.

## 2 Materials and methods

### 2.1 Yeast strains and culture

*C. albicans* ATCC 10231 strain obtained from the American Type Culture Collection (Manassas, VA, USA) was cultured to the yeast-like form in YPD medium (1% yeast extract, 2% soybean peptone, and 2% glucose) (Sigma, St. Louis, MO, USA) at 30°C for 16 h on an orbital rotary shaker MaxQ 6000 (180 rpm) (Thermo Fisher Scientific, Waltham, MA, USA) until it reached the stationary phase. Cell numbers were determined by optical density (OD) measurements at 600 nm.

### 2.2 Biofilm formation

To isolate eDNA and eRNA from ECM, biofilm was obtained by the inoculation of the stationary phase cells (10^8^ cells/1ml) into 20 mL of the RPMI-1640 medium (PAA Laboratories GmbH, Pasching, Austria), followed by further incubation with constant shaking at 37°C for 48 h (170 rpm) in a 200 ml Erlenmeyer flask.

### 2.3 Fungal nucleic acid isolation

A pool of extracellular nucleic acids was obtained according to the method described previously (12). Briefly, *C. albicans* biofilms were cultured on the surface of Erlenmeyer flasks, in 20 ml of RPMI medium activating filamentation, for 48 hours at 37°C. Then the biofilms were scratched, and the pellet was suspended in 100 mM Tris-HCl buffer with 0.9% NaCl pH 7.4, and then washed: with 100 mM Tris-HCl buffer with 0.9% NaCl pH 7.4, and 50 mM Tris-HCl buffer pH 7.5. The solutions of ECM components were prepared by gentle enzymatic degradation with β-1,3-glucanase (Lyticase from Arthrobacter luteus; Sigma-Aldrich, St. Louis, MO). Lyticase (1250 U per 1** **g of biofilm wet mass) was added to the cell suspension in 2** **ml of 50 mM Tris buffer, pH 7.5 containing 40 mM β-mercaptoethanol and protease inhibitors (Roche, Penzberg, Germany), and incubated for 2** **h at 37°C with shaking (170 rpm). After a series of centrifugations, the supernatant was dialyzed against 50 mM Tris-HCl pH 7.5. Extracellular nucleic acids were separated from the cell wall proteins (CWP) by ion-exchange chromatography (IEC) on MonoQ-Sepharose column (GE Healthcare/Pharmacia, Uppsala, Sweden) using the gradient described in a previous publication (12). In addition, nucleic acids were also obtained from the entire biofilm. For this purpose, *C. albicans* cells from a 48** **h culture in RPMI-1640 were scraped from the sides of the flask, the suspension placed in an Eppendorf tube, centrifuged and washed twice with PBS buffer. Then, sterile glass beads (425-600 μm), 200 μl of lysis buffer (2% Triton X-100, 1% SDS, 100 mM NaCl, 10 mM Tris-HCl, pH 8, 1 mM EDTA) and 200 μl phenol: chloroform:isoamyl alcohol (25:24:1) were added to the cell pellet and samples were vortexed for 2 minutes. The cell homogenization process was carried out using the Bertin Precellys Evolution cell homogenizer (Bertin Instruments, Montigny-le-Bretonneux, France). Then, 200 μl of TE buffer (10 mM Tris-HCl, pH 8, 1 mM EDTA) was added to the homogenate. The lysed samples were centrifuged (20** **min, 13000 x g, 4°C). An equal volume of chloroform was added to the aqueous phase of homogenate and centrifuged (20** **min, 13000 x g, 4°C). To precipitate DNA, 2 volumes of 96% ethanol were added to the aqueous phase, then the samples were incubated overnight at -20°C. After incubation, samples were centrifuged (15** **min, 12000 x g, 4°C). The white precipitate containing DNA was washed twice with 70% ethanol and dried, then dissolved in 100 μl of nuclease-free water. Total RNA isolation performed similarly to DNA, except that TRI-Reagent (Merck, Darmstadt, Germany) was used instead of phenol:chloroform:isoamyl alcohol (25:24:1). Sample purity was assessed using an absorbance measurement in the range from 240 nm to 300 nm with a Biotek Synergy H1 microplate reader (BioTek Instruments, Winooski, VE, USA).

### 2.4 Preparation of nucleic complex with LL-37, LL-25, and LL-8

Human LL-37 (LLGDFFRKSKEKIGKEFKRIVQRIKDFLRNLVPRTES) was obtained from Sigma-Aldrich (St. Louis, MO). The shorter derivatives formed by the action of the Saps proteases (LL-25: LLGDFFRKSKEKIGKEFKRIVQRIK; LL-8: LLGDFFRK) were obtained as previously described (4). For standardization of optimal formation of complexes, nucleic acids and peptides were incubated at different ratios in PBS at room temperature (RT). The formation of complexes and their stability was confirmed by the electrophoretic mobility shift assay (EMSA), using 1% agarose gel. Finally, in all experiments, 0.5 µg of DNA/RNA was mixed in 1 µg of LL-37/LL-25/LL-8 peptides in 1 ml of Dulbecco’s Modified Eagle’s Medium (DMEM, Gibco, USA) and added to neutrophils in 100 µl or 500 µl, depending on the type of analysis.

### 2.5 Neutrophil isolation

Neutrophils were isolated from EDTA-treated (5 mM) whole-blood samples obtained from healthy, anonymous donors via the Regional Blood Donation Center (Cracow, Poland), which complies with the requisite confidentiality assurances for human participants. The blood samples were centrifuged at 300 g for 20 min to remove the plasma layer. Then 2/3 of the upper phase containing the plasma was discarded and the remaining suspension was diluted with Mg^2+^- and Ca^2+^-free PBS buffer (Biowest, Nuaille, France). The suspension was overlaid on the lymphocyte separation medium (Biowest, Nuaille, France) and centrifuged (420 g for 30 min.). The low-density fractions were discarded. The lower phase, containing RBCs and granulocytes were diluted in 1% polyvinyl alcohol. The separation of erythrocytes from granulocytes was carried out for 20 min, after which the upper phase, containing granulocytes, was collected into a new tube, and centrifuged at 420 x g for 5 minutes. Residual RBCs were lysed by 1 ml of Red Blood Lysis Buffer (Roche, Penzberg, Germany). The granulocyte pellet was suspended in 1 ml DMEM (Gibco, USA). The number of cells was determined using a Bürker chamber. The neutrophil purity was assessed routinely by forward- and side-scatter flow cytometric analyses. The applied isolation method yields a >95% pure population of the cells.

### 2.6 Endocytosis analysis

Yeast nucleic acids were labeled with the Ulysis™ Alexa Fluor™ 546 Nucleic Acid Labeling Kit according to the manufacturer’s instructions. For microscopic observations, neutrophils (8x10^5^/well) were plated on a 96-well plate with #1.5 glass-like polymer coverslip bottom (Cellvis, Mountain View, CA). Labeled DNA/RNA and their complexes with the LL-37 peptide were then added to the cells. The progress of endocytosis was observed by fluorescence microscopy (IX73, Olympus). The obtained microscopic images were processed in the Olympus cellSens Dimension 3.1 imaging software.

### 2.7 Chemotaxis analysis

The chemotaxis of neutrophils was evaluated by the 12-well chemotaxis chamber technique (Transwell^®^-Clear inserts, Corning, NY, USA). 10^6^ cells/ml neutrophil were applied to the inserts (3 µm pores). Tested samples were placed in the lower compartment of the chamber. Neutrophils were incubated at 37°C in 5% CO_2_ atmosphere for 1 h. The cells that migrated into the lower compartment of the chamber were stained with CellTracker™ Green (Invitrogen, Waltham, MA) and counted in ImageJ software.

### 2.8 NET visualization and quantification

Neutrophils (10^6^ cells/ml) were seeded in the well of the 96-wells microplate (Greiner Bio-One, Frickenhausen, Germany), in 100 µl of DMEM (Gibco, USA) and incubated for 20 min at 37°C, 5% CO_2_ to enable attachment to the surface. The stimuli factors including: fungal DNA, RNA, DNA:LL-37, DNA:LL-25, DNA:LL-8, RNA:LL-37, RNA:LL-25, RNA:LL-8 were added to cells in 100 µl DMEM (Gibco, USA), at a selected range of concentrations. Phorbol 12-myristate 13-acetate (PMA) at final concentration of 25 nM was used as a positive control. The stimulation was carried out for 3 hours at 37°C, 5% CO_2_. For fluorescence microscopy visualization, after stimulation neutrophils were stained with Sytox Green and the released NETs were visualized using a fluorescence microscope (IX73, Olympus). For the NETosis quantification, the factors were removed after incubation, and 100 µl aliquot of micrococcal nuclease MNase (1U/ml) was added to the wells and incubated for an additional 20 min at 37°C. The supernatants were centrifuged and stained with Sytox Green fluorescence dye (final concentration 1 μM). 80 μl of the samples were transferred into a 96-well microplate and the fluorescence was measured using the microplate reader (excitation: 495 nm, emission: 525 nm).

### 2.9 Analysis of ROS production

Dihydrorhodamine 123 (DHR 123; Invitrogen, Waltham, MA) was used to detect ROS production. Neutrophils 10^6^/ml were seeded in a 96-well plate in 100 μl DMEM (DMEM (Gibco, USA) or placed in an Eppendorf tube in 500 μl DMEM. Then, DHR123 (final concentration 5 μM) was added for 10 minutes and cells were washed with PBS. Neutrophils were incubated with 100 µl or 500 µl of the stimulating factors for 1 h at 37°C, 5% CO_2_. Cells stimulated by PMA (25 nM) were used as the positive control. The fluorescence of oxidized rhodamine 123 was measured using a microplate reader (H1, Biotek), or flow cytometry (BD Fortessa).

### 2.10 Apoptosis activation assays

Neutrophils stimulated by selected factors were stained with propidium iodide (PI) and fluorescein isothiocyanate (FITC)-labeled Annexin V (AnV) (Dead Cell Apoptosis Kit with Annexin V-FITC and PI, Invitrogen, Carlsbad, CA, USA), according to the supplier’s instruction. The analysis of apoptosis progress was performed using flow cytometry (LSR Fortressa, BD, San Jose, CA, USA). In the second way, neutrophils were stained with the CellEvent™ Caspase-3/7 Detection Reagents kit (Invitrogen, Waltham, MA). The fluorescence was measured on a microplate fluorescence reader (H1, Biotek) - excitation: 495 nm, emission: 525 nm.

### 2.11 Mcl-1 protein analysis

Neutrophils (10^6^/ml) were seeded in the well of 12-wells plate (in 500 μl of DMEM) and incubated for 20 min at 37°C, 5% CO_2_ to enable attachment to the surface. Then, 300 ul of stimulating factors were added and incubated for 2 hours at 37°C. After incubation, neutrophils were lysed using a cold RIPA buffer (10 mM Tris-HCl, pH 8.0; 1 mM EDTA; 0.5 mM EGTA; 1% Triton X-100; 0.1% Sodium Deoxycholate; 0.1% SDS; 140 mM NaCl) supplemented with protease and phosphatases cocktail inhibitors (Merck, Merck, Darmstadt, Germany). The lysates were incubated on ice for 30 minutes and then centrifuged at maximum speed to collect the supernatant containing total protein. Protein concentrations were determined using a Pierce™ BCA Protein Assay Kits (Thermo Fisher Scientific, Waltham, MA, USA). Equal amounts of total protein (20 µg) from each sample were mixed with Laemmli buffer and heated at 95°C for 5 minutes. Samples were loaded onto 12% polyacrylamide gels and subjected to sodium dodecyl sulfate-polyacrylamide gel electrophoresis (SDS-PAGE). Proteins separated by SDS-PAGE were transferred onto nitrocellulose or PVDF membranes using wet electroblotting systems (BioRad, Hercules, CA, USA). Membranes were blocked for 1 h with 5% non-fat milk in 20 mM Tris-Buffered Saline with 0.1% Tween-20 (TBST) to reduce non-specific binding. Membranes were incubated overnight at 4°C with a rabbit primary antibody anti-Mcl-1 (Abcam, Cambridge, UK) at dilution recommended by the manufacturer (1:1000). Antibodies anti-β-actin (Cell Signaling Technology, Danvers, Massachusetts, USA) were used as a loading control. After incubation, membranes were washed three times with TBST and then incubated with a horseradish peroxidase (HRP)-conjugated secondary antibody (R&D System, Minnesota, USA) at room temperature for 1 hour. Protein bands were visualized using a chemiluminescence detection system (ChemiDoc Imaging Systems, BioRad). The intensity of Mcl-1 bands was quantified using ImageJ, and the results were normalized to the loading control, providing relative protein expression levels.

### 2.12 Cytokines analysis

Neutrophils (10^6^ cells/ml) were seeded on 24-well plate (Corning, NY, USA) and stimulated with 300 ul of DNA/RNA samples and its complex with LL-37 peptide for 3h. Cells stimulated with LPS (100 ng/ml) served as positive control. After stimulation, supernatants were collected and centrifuged (300 x g) to remove cells debris. Cytokines levels were then determined using an BD OptEIA™ Human IL-8, IL-6 and IL-1β ELISA kits (BD Biosciences, NY, US).

### 2.13 Statistical analysis

Statistical analysis was performed with the GraphPad Prism 9 software (GraphPad Software, CA, USA). The results are presented as a mean with a standard error of the mean (SEM). To assess the significance between groups, a t-test or one-way ANOVA with Dunnett’s *post-hoc* test was performed. The results were considered statistically significant at p-value <0.05 (* - p <0.05, ** - p <0.01, *** - p <0.005, **** - p <0.001) and statistically insignificant (ns) for p> 0.05.

## 3 Results

### 3.1 LL-37 facilitates endocytosis of *C. albicans* nucleic acids

**Figure 1:**
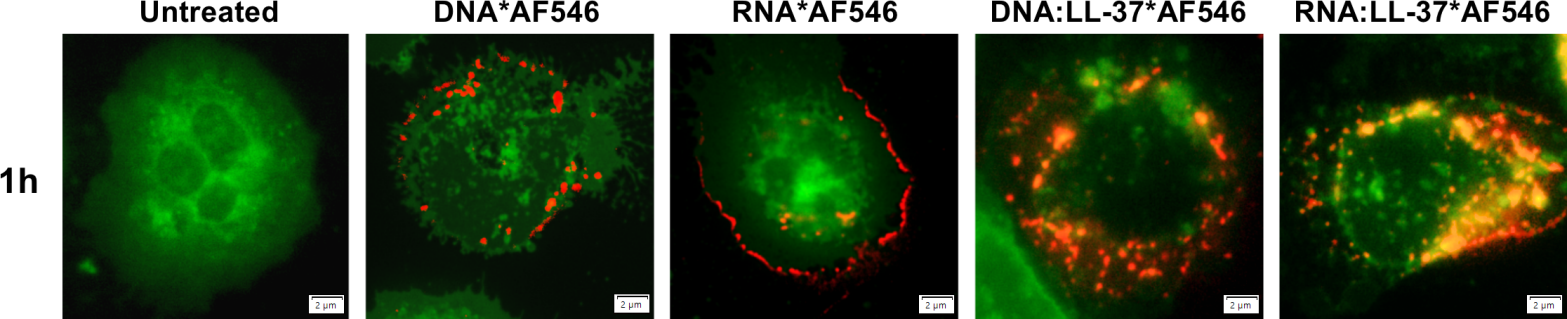
The presence of LL-37 drives the intracellular accumulation of yeast nucleic acids. To track the internalization of DNA and RNA and their complexes with LL-37, fungal nucleic acids were labeled with ULYSIS™ Alexa Fluor (AF) 546 reagent and purified by Micro Bio-Spin™ P-30 Gel Columns according to manufacturer instruction. The surface of the neutrophils was marked with CellMask™ Alexa Fluor 488 Plasma Membrane Stains (dilution 1:1000). Neutrophils (5x10^6^/ml in 100 μl DMEM) were incubated with the complexes for 1 h at 37°C, 5% CO_2_. The samples were analyzed microscopically (Olympus IX73) in the FITC and mCherry channels corresponding to the dyes used. Image deconvolution was performed in the CellSens 3.1 software. The figure shows representative images in merged channels. The scale bar on the images is 2 μm.

The negative charge of nucleic acids resulting from the presence of phosphate residues significantly hinders the efficient endocytosis of these molecules by immune cells. Hence, in the initial phase of research, we checked whether the presence of positively charged LL-37 peptide contributes to the facilitation of yeast nucleic acid endocytosis. The concentration of LL-37 at the site of an infection may vary significantly depending on the severity of the infection. In plasma, the level of LL-37 may oscillate approximately 0.5-3 µg/ml (41). In our previous publication, we showed that *in vitro*, neutrophils are activated by relatively low concentrations of eDNA and eRNA, below 1 µg/ml (12). Based on this information, we conducted optimization to establish conditions for efficient complex formation. For this purpose, complexes were generated by incubating LL-37 and fungal DNA/RNA at different mass ratios (**Supplementary Figure 1A**). In order to find the optimal ratio, an EMSA test was carried out. A distinct effect of complex formation was observed with a two-fold mass excess of LL-37. Based on these results, we selected an initial concentration of 0.5 µg/ml for both DNA and RNA and 1 µg/ml LL-37 for complex formation in all experiments. In order to visualize the *in-vitro* generated DNA/RNA:LL-37 or free DNA, RNA molecules, we used the Ulysis™ Alexa Fluor™ 546 kit, which enables fast coupling of fluorescent dye to purine bases in nucleic acid polymers. Samples were purified on Micro Bio-Spin™ P-30 Gel Columns to eliminate any unbound dye. This methodological approach was employed to prevent nonspecific labeling of human nucleic acids, including those residing within the cellular nucleus. The neutrophil cell membrane was labeled with CellMask™ Alexa Fluor™ 488 dye. The endocytosis process was monitored by fluorescence microscopy for up to 1 hour. The obtained results clearly indicate that the formation of complexes with the LL-37 plays a pivotal role in driving the intracellular accumulation of *C. albicans* DNA and RNA molecules. In contrast, those nucleic acid molecules not engaged in complexation with LL-37 exhibit significantly delayed cellular uptake and primarily localize within the cellular membrane (**Figure 1**).

### 3.2 DNA:LL-37 and RNA:LL-37 complexes are a chemotactic factors

**Figure 2:**
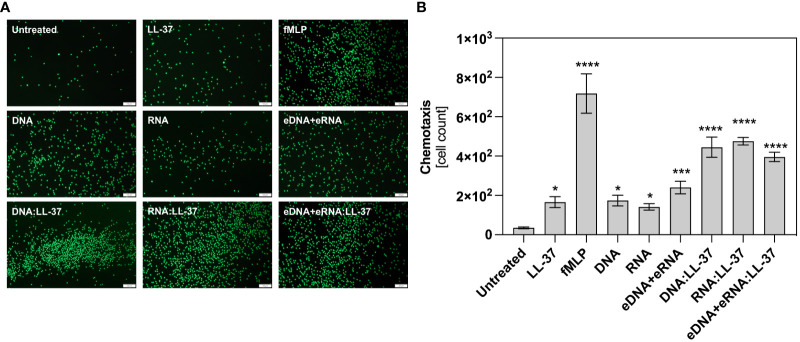
DNA/RNA:LL-37 complexes drive neutrophil chemotaxis. The effect of DNA:LL-37 and RNA:LL-37 on neutrophil chemotaxis was verified using the Transwell migration assay. 10^6^ cells/well were applied to the inserts (3 µm) and tested samples: LL-37 – 1 μg/ml, fMLP – 1 μM, DNA, RNA, eDNA+eRNA – 0.5 μg/ml, and complexes DNA/RNA (0.5 μg/ml) + LL-37 (1 μg/ml) were placed in the lower compartment of well. After 1 hour of incubation the cells migrated through the membrane were stained with Cell Tracker Green, imaged using Olympus IX73 microscope **(A)** and counted in ImageJ software **(B)**. The graph shows a representative result of the quantitative analysis. To assess significance one-way ANOVA with Dunnett’s multiple comparisons *post-hoc* test was performed. The results were considered statistically significant at p-value <0.05 (* - p <0.05, *** - p <0.005, **** - p <0.001). The scale bar on the images is 100 μm.

In the next stage of the research, we assessed the impact of LL-37 complex formation with yeast nucleic acids on neutrophil chemotaxis. For this purpose, we employed a Transwell^®^ membrane assay as previously described (12). The tested agents, at optimized concentrations, were added to the bottom of the chamber. Neutrophils were added to the inserts with porous membrane, which enables transmigration towards the chemoattractant. The negative control contained neutrophils in contact with the DMEM medium. N-formylmethionine-leucyl-phenylalanine (fMLP), a well-known chemotactic agent of bacterial origin, was used as positive control. After 1 hour of incubation, the number of fluorescently labeled cells at the bottom of the chamber was counted using ImageJ software. The obtained results indicate that the formation of DNA/RNA:LL-37 complexes enhance (2-fold) the chemotaxis of neutrophils. Representative results are shown in **Figure 2**.

### 3.3 The formation of fungal DNA/RNA complexes with LL-37 and its shorter derivatives attenuates the activation of NETosis

**Figure 3:**
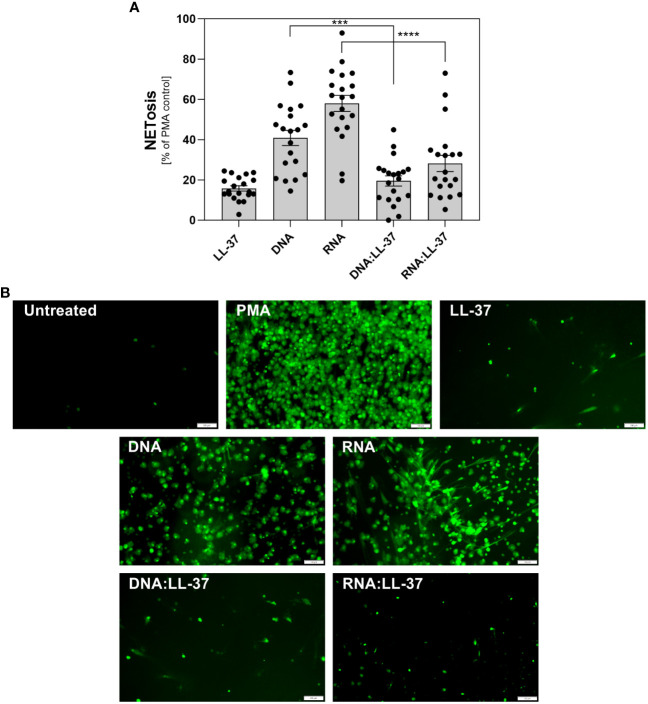
The role of DNA:LL-37 and RNA:LL-37 complexes in NETosis activation. Neutrophils (10^6^ cells/ml) were incubated at 37°C for 3 hours with 100 μl DMEM (negative control), PMA (25 nM, positive control), LL-37 (1 μg/ml), DNA (0.5 μg/ml), RNA (0.5 μg/ml), and complexes DNA/RNA 0.5 μg/ml + LL-37 1 μg/ml. **(A)** After stimulation, NETs were stained with Sytox Green (final concentration 1 μM) and quantified after 20 minutes of treatment with Mnase (1U/ml). The results depicted in the graphs are expressed as a percentage relative to the responses elicited by PMA, the response of unstimulated neutrophils was considered as 0%. Data represent a comparison of NETosis activation for 20 independent experiments ± SEM. To assess statistical significance ANOVA with Dunnett’s multiple comparisons *post-hoc* test was used. The results were considered statistically significant at p-value <0.05 (ns > 0.05, *** - p <0.005, **** - p <0.001). **(B)** NETs were stained with Sytox Green dye (final concentration 1 μM) and visualized using Olympus IX73 fluorescence microscopy. The scale bar on the images is 100 μm.

Upon activation of neutrophils by biofilm components, significant amounts of antimicrobial peptides, including LL-37, may be released. The partial complexation of nucleic acids present in the extracellular matrix (ECM) by LL-37 may influence the immune response of these cells. To investigate this hypothesis, we compared the levels of NETosis activation by free DNA/RNA molecules and by their complexes with LL-37. As the nucleic acids isolated from the *C. albicans* ECM consist of both eDNA and eRNA and because neutrophil responses can vary depending on the type of nucleic acid, we conducted experiments using purified fungal DNA and RNA acquired from whole biofilm-forming cells (12). We performed 20 independent experiments using neutrophils isolated from healthy donors (**Figure 3A**). In each experiment, neutrophils were incubated with the tested factors for a maximum of 3 hours. The concentration of nucleic acids was selected based on prior research, which identified the most efficient response at a concentration of 0.5 μg/ml. Neutrophils, chemically activated with phorbol 12-myristate 13-acetate (PMA) were used as a positive control (100%) for NETosis activation. The concentration of LL-37 used for controls and complexing was optimized to 1 μg/ml in previous steps. Our findings revealed that neutrophil stimulation with DNA:LL-37 complexes resulted in a reduced level of NETosis, dropping from approximately 40% of the positive control (PMA) observed for free DNA treatment to approximately 20%. A similar correlation was observed for RNA:LL-37, though with a considerably larger decrease in NETosis activation (approximately 3-fold). The results depicted in the graphs are expressed as a percentage relative to the responses elicited by PMA, with the response of unstimulated neutrophils considered as 0%. To identify the NETs structures, the Sytox Green dye was used, and the progress of the NETosis was also monitored by fluorescence microscopy (**Figure 3B**).

**Figure 4:**
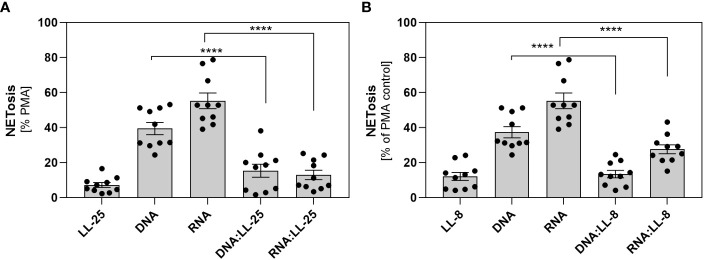
The role of shorter LL-37 derivatives in NETosis activation. Neutrophils (10^6^ cells/ml) were incubated with 100 μl DMEM (negative control), PMA (25 nM), LL-25/LL-8 (1 μg/ml), DNA (0.5 μg/ml), RNA (0.5 μg/ml), and complexes DNA/RNA 0.5 μg/ml + LL-25/LL-8 – 1 μg/ml. **(A)** After stimulation, NETs were stained with Sytox Green (final concentration 1 μM) and quantified after 20 minutes of treatment with Mnase (1U/ml). Results for the complexes with LL-25 are presented in **(A)**, and for LL-8 in **(B)**. Data represent a comparison of NETosis activation for 10 independent experiments ± SEM. To assess statistical significance ANOVA with Dunnett’s multiple comparisons *post-hoc* test was used. The results were considered statistically significant at p-value <0.05 (**** - p <0.001).

In our previous research, we demonstrated that *C. albicans* can partially neutralize the activity of the LL-37 peptide by enzymatic degradation, resulting in the formation of its shorter derivatives (4). Despite the partial reduction in its antimicrobial efficacy, these fragments retain a positive charge and can form electrostatic complexes with fungal nucleic acids (**Supplementary Figures 1B, C**). In the next step, we checked whether the presence of shorter LL-37 fragments could also influence the neutrophil response, as observed with the native peptide. In our experiments, we employed LL-8, a fragment generated by the action of Sap1 and Sap4, and LL-25 produced through the action of Sap2, Sap3, Sap8, and Sap9. The experiments were performed on samples from 10 independent donors. The results obtained closely paralleled those observed with the native LL-37 peptide, indicating the inhibition of NETosis irrespective of the peptide’s length (**Figures 4A, B**).

### 3.4 DNA:LL-37 and RNA:LL-37 complexes inhibit PMA-activated and ROS-dependent NETosis

**Figure 5:**
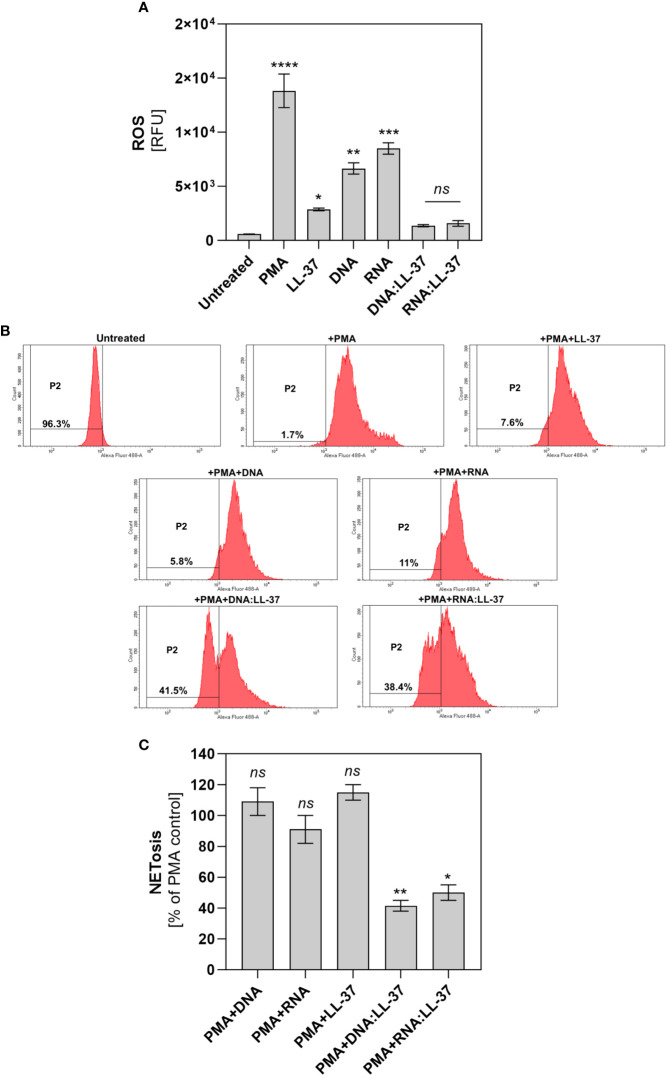
Effect of DNA:LL-37 and RNA:LL-37 complexes on the ROS-dependent pathway. **(A)** Neutrophils (10^6^ cells/ml) were stained with DHR 123 (final concentration 5 μM), then washed with PBS and incubated with LL-37 (1 μg/ml), DNA (0.5 μg/ml), RNA (0.5 μg/ml), and complexes DNA/RNA 0.5 μg/ml + LL-37 – 1 μg/ml for 1h. The fluorescence of oxidized DHR 123 was measured using a BioTek Synergy H1 microplate reader (excitation: 495 nm, emission: 525 nm). **(B)** Neutrophils (10^6^/sample) were stained with DHR 123 (5 μM) and then 500 µl of stimulants were added to them for 30 minutes (analogous to points A). After this time, 10 μl PMA (to final concentration 25 nM) was added to the samples, and after 30 minutes the ROS level was measured on a flow cytometer (BD Fortessa). The data represent the histograms obtained for 10,000 neutrophils in Alexa Fluor 488 channel. **(C)** Neutrophils (10^6^ cell/ml) were incubated with LL-37 (1 μg/ml), DNA (0.5 μg/ml), RNA (0.5 μg/ml), and complexes DNA/RNA 0.5 μg/ml + LL-37 – 1 μg/ml for 30 minutes, then PMA was added to the cells (final concentration 25 nM). After 2-hour incubation, the released NETs were stained with Sytox Green dye (1 μM) and quantified using Mnase (1U/ml). The data in the graph was normalized: 0% - unstimulated control and 100% - PMA. Representative results ± SEM are shown in the graph. To assess statistical significance ANOVA with Dunnett’s multiple comparisons *post-hoc* test was used. The results were considered statistically significant at p-value <0.05 (ns > 0.05, * - p <0.05, ** <0.01, *** - p <0.005, **** - p <0.001). RFU, relative fluorescence unit.

In the next step, we assess the impact of DNA:LL-37 and RNA:LL-37 on ROS generation. To accomplish this, neutrophils were stained with dihydrorhodamine 123 (DHR 123), a widely used dye, that can passively diffuse across membranes where it undergoes oxidation by ROS to its cationic, fluorogenic form. The results obtained indicate a notably weaker ROS generation following activation with DNA:LL-37 and RNA:LL-37 compared to free DNA, RNA, or LL-37 peptide (**Figure 5A**). Next, we investigated whether the complexation of fungal nucleic acids affects the PKC-dependent ROS generation pathway, which is typically activated by PMA. For this purpose, neutrophils were pre-stimulated for 30 minutes with DNA/RNA and *in vitro* generated complexes with LL-37. Subsequently, PMA was added to each sample and incubated for 1 hour. The level of ROS generation was quantified using flow cytometry. The obtained results (**Figure 5B**) revealed that 30 minutes of stimulation with DNA:LL-37 and RNA:LL-37 induces the formation of a population of neutrophils (in gate P2 – 40%) with completely inhibited ROS production and not responding to PMA. In the case of the other samples, we did not observe noticeable change. Then, we tested whether DNA:LL-37 and RNA:LL-37 stimulation would block the generation of NETs via the ROS-dependent pathway induced by PMA. For this purpose, neutrophils were pre-stimulated as described above and then PMA was added for 2 hours. After this time, the level of NETs generation was quantified. The complexation of yeast nucleic acids significantly impairs PMA-induced NETosis, while this response remains unaffected by other stimulating factors (**Figure 5C**). Nevertheless, it should be noted that, as in the case of ROS detection, the presence of a population of cells forming NETs is observed, which activates NETosis at a level of approximately 40% of PMA.

### 3.5 Neutrophil activation by fungal nucleic acid complexes with LL-37 prolongs neutrophil lifespan

**Figure 6:**
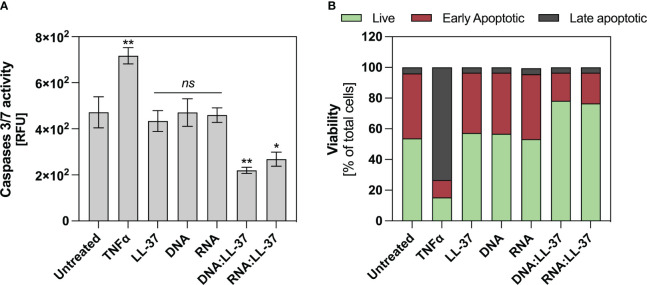
Anti-apoptotic effect of DNA/RNA:LL-37 complexes. **(A)** Neutrophils (10^6^/ml) were incubated with TNFα - 100 ng/ml, LL-37 – 1 μg/ml, DNA, RNA, and DNA/RNA complexes 0.5 μg/ml + LL-25/LL-8 – 1 μg/ml in 100 μl of DMEM for 2 hours. Then, the stimulating factors were removed, and the cells were labeled with CellEvent™ Caspase-3/7 Detection Reagents (Invitrogen™) according to the manufacturer’s instruction. Then, after 30 minutes of incubation, the fluorescence level was measured using a Biotek H1 microplate reader (excitation: 495 nm, emission: 525 nm). To assess statistical significance ANOVA with Dunnett’s multiple comparisons *post-hoc* test was used. The results were considered statistically significant at p-value <0.05 (ns > 0.05, ** <0.01, * - p <0.05). RFU, relative fluorescence unit. **(B)** Neutrophils (10^6^/sample) were incubated with stimulating factors at the same concentrations as in point A, but in 500 μl DMEM. Cells were then labeled with Dead Cell Apoptosis Kits with Annexin V and PI for Flow Cytometry (Invitrogen™) according to the manufacturer’s protocol. Then, the cells were analyzed cytometrically in the green (Annexin V) and red (PI) channels. The graph shows the percentage of alive (green), early apoptotic (red), late apoptotic (grey) for a representative result for 10.000 cells.

Neutrophils exhibit a very short lifespan in the bloodstream, nevertheless, their viability experiences dynamic alterations upon stimulation. Exposure to a potent stimulatory agent may lead to a delay in apoptosis (42). Considering that elevated intracellular ROS levels can trigger apoptotic signaling cascades and the subdued ROS generation upon exposure to DNA:LL-37 and RNA:LL-37, this result suggest that these molecules might have an anti-apoptotic effect. In order to verify the impact of the DNA:LL-37 and RNA:LL-37 complexes on the viability of neutrophils, the cells were incubated for 2 hours with stimulating factors. The enzymatic activities of caspases 3 and 7 were assessed using specific fluorescent substrates. As a positive control for apoptosis induction, neutrophils treated with TNFα were employed. The data suggest that stimulation with DNA:LL-37 and RNA:LL-37 complexes do not result in increased activation of pro-apoptotic caspases; contrarily, their activities remain at a lower level than in unstimulated control (**Figure 6A**). Subsequently, we performed the annexin V and propidium Iodide (PI) cell viability assay. The obtained flow-cytometry results indicate the presence of about 30% more cells with a non-apoptotic phenotype when contacted with the complexes than in the case of stimulation with free DNA, RNA and LL-37 molecules (**Figure 6B**).

**Figure 7:**
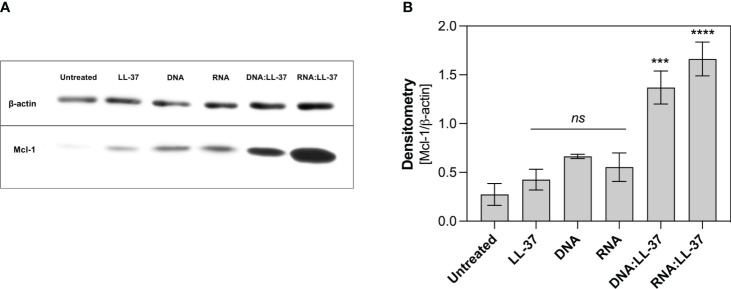
Analysis of the level of anti-apoptotic protein Mcl-1. Neutrophils (10^6^/ml) were plated in a 12-well plate and stimulated with LL-37 (1 μg/ml), DNA, RNA (0.5 μg/ml), and DNA/RNA complexes + LL-37 for 2 hours. Protein levels were then analyzed by Western Blot. **(A)** Representative image of membrane **(B)** Relative levels of Mcl-1 protein were quantified by densitometry and normalized to β-actin. The graph shows the normalized values obtained for 3 independent experiments ± SEM. To assess statistical significance ANOVA with Dunnett’s multiple comparisons *post-hoc* test was used. The results were considered statistically significant at p-value <0.05 (ns > 0.05, *** - p <0.005, **** - p <0.001).

One key player in the regulation of neutrophil survival is the Myeloid Cell Leukemia-1 (Mcl-1) protein. Mcl-1 belongs to the Bcl-2 family of proteins, known for their pivotal role in modulating apoptosis (42, 43). Mcl-1 is particularly abundant in neutrophils and exerts a potent anti-apoptotic effect by preventing the activation of mitochondrial apoptotic pathways (42). In the subsequent step, we aimed to ascertain whether the DNA:LL-37 and RNA:LL-37 complexes could lead to an increase in Mcl-1 levels. To investigate this, neutrophils were incubated for a 2-hour stimulation period and protein levels were assessed using Western Blot and specific antibodies. The performed analyzes clearly showed a significant increase in the production of Mcl-1 after stimulation with DNA:LL-37 and RNA:LL-37 complexes (**Figure 7**). In most experiments, a slightly greater increase in Mcl-1 was observed in response to RNA:LL-37 stimulation. This result effectively supports previous observations and confirms the anti-apoptotic effect of fungal nucleic acid complexed by LL-37 peptide.

### 3.6 The extended lifespan of neutrophils promotes the production of IL-8 in response to DNA/RNA:LL-37 complexes

**Figure 8:**
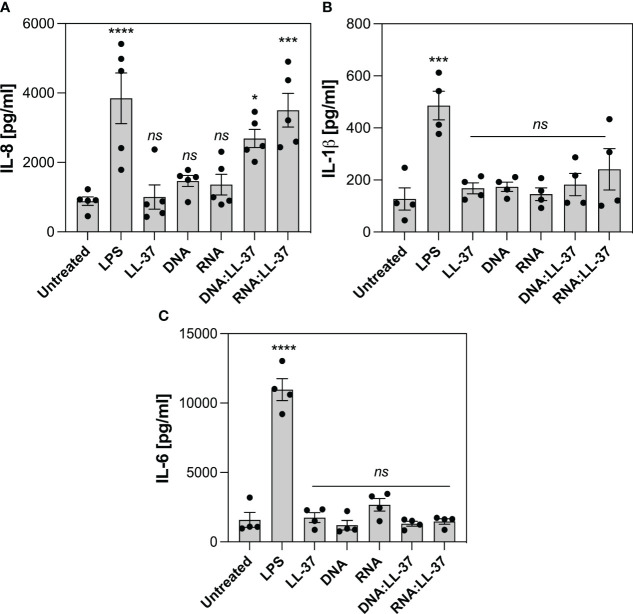
Effect of DNA:LL-37 and RNA:LL-37 complexes on the neutrophil cytokine response. The graphs illustrate the levels of IL-8 **(A)**, IL-1β **(B)** and IL-6 **(C)** production by neutrophils following exposure to DNA:LL-37 and RNA:LL-37 complexes. Neutrophils (10^6^/ml) were seeded on 24-well plate and incubated with 300 μl stimulating factors: LPS (100 ng/ml), LL-37 (1 μg/ml), DNA, RNA, and DNA/RNA complexes (0.5 μg/ml + LL-37 1 μg/ml) for 3 hours. The concentration of cytokines in supernatants was quantified using dedicated ELISA kits (BD Biosciences, NY, US). The results were considered statistically significant at p-value <0.05 (* - p <0.05, *** - p <0.005, **** - p <0.001) and statistically insignificant (ns) for p> 0.05 (ANOVA with Dunnett’s multiple comparisons *post-hoc* test).

Despite neutrophils possess 10-20 times less RNA than other leukocytes, they actively participate in the synthesis of cytokines that belong to both pro-inflammatory and anti-inflammatory classes (44). The production of cytokines by neutrophils at the site of infection plays a pivotal role in coordinating the immune response and aiding in the clearance of pathogens. In the last phase of the experiments, we investigate whether the exposure to DNA:LL-37 and RNA:LL-37 complexes is associated with increased production of IL-8, IL-1β and IL-6. During this phase of the experiment, neutrophils were incubated with appropriate factors for 3 hours, after which the level of cytokines was determined using the ELISA method. LPS derived from *Escherichia coli* was used as a positive control for cytokine activation. Our research findings indicate a significant increase in IL-8 production by neutrophils in response to both DNA:LL-37 and RNA:LL-37 treatments (**Figure 8A**). We did not detect any statistically significant alterations in the levels of IL-1β and IL-6 production (**Figures 8B, C**).

## 4 Discussion

Neutrophils play a crucial role in the host’s defense against fungal infections, including those caused by *C. albicans*. These innate immune cells are often the first responders to invading fungal pathogens and contribute significantly to the overall immune response. Their multifaceted functions, from endocytosis to the release of antimicrobial agents and the formation of NETs, underscore their importance in containing and ultimately eliminating fungal pathogens like *C. albicans*. While significant progress has been made in the area of immune responses to *C. albicans*, several aspects of this intricate process remain unclear, particularly in the context of response to contact with fungal biofilm. Some studies suggested that the formation of biofilms by *C. albicans* gradually modifies neutrophil activity, leading to the suppression of NETosis and endocytosis (28, 45). However, the exact mechanisms responsible for this phenomenon are not entirely clear, and it is probable that multiple factors contribute to this observed alteration (28, 45).

In this study, we have demonstrated for the first time, that mutual interactions between neutrophil and fungal-derived factors can influence the observed changes in neutrophil response to biofilm-forming infection. Specifically, our focus has been directed on the interactions between the LL-37 peptide and nucleic acids which may be present within the matrix of *C. albica*ns biofilm. The LL-37 peptide has gained considerable scientific attention in recent years due to its broad spectrum of immunomodulatory functions (46). However, its role in modulating the response of neutrophils to fungal nucleic acids has not yet been investigated, especially in the context of *C. albicans* infections.

In our current study, firstly we evaluated the impact of the LL-37 peptide on the efficiency of *C. albicans* nucleic acids endocytosis. The obtained results indicate non-specific electrostatic interactions between LL-37 and nucleic acids result in the neutralization of the negative charge of DNA and RNA, thereby facilitating their transport across the plasma membrane and leading to their rapid accumulation inside the cell. The exact transport mechanism remains unclear. In studies carried out by Sandgren et al. on the CHO-K1 model, it was shown that the transport of plasmid DNA derived from *E. coli* in complex with LL-37 may involve receptor-independent lipid raft-mediated endocytosis (32). Similar conclusions were obtained in the case of studies on monocytes, conducted in the context of self-DNA recognition (47).

LL-37 exhibits chemotactic activity towards leukocytes and this activity is likely mediated through interactions with formyl-peptide receptor (FPR) (48). The chemotactic effect was also observed in the case of fungal nucleic acids, as we previously showed (12). Analysis of the impact of DNA:LL-37 and RNA:LL-37 complex formation on the chemotaxis process revealed a significant intensification of the chemotactic effect, compared to free molecules. These findings suggest that the formation of DNA:LL-37 and RNA:LL-37 complexes enable immune cells to swiftly locate and target the biofilm-forming yeasts, aiding in the early containment and elimination of the infection. This enhances chemotactic response compared to free LL-37, may suggest the involvement of additional surface receptors, but further studies are required.

To date, research has revealed that numerous *C. albicans* virulence factors activate the generation of NETs, using different pathways and engaging various receptors. These factors include primarily cell wall polysaccharides (β-glucans, mannans), secreted aspartyl proteases and quorum-sensing molecules (22, 23, 49). In our previous research, we demonstrated that extracellular nucleic acids, a constituent of the ECM of biofilm, also serve as potent triggers for NETosis, especially when present in relatively low concentrations (12). Due to the observed enhanced phagocytic and chemotactic abilities, we investigated whether the formation of DNA:LL-37 and RNA:LL-37 complexes also affect the activation of NETosis. The results indicated a significant inhibition of NETosis activation during stimulation with DNA-LL-37 and RNA:LL-37 complexes. These observations suggest, that LL-37 and NETs are probably released into the extracellular space as part of the early immune response to fungal infection. As the infection progresses, the increasing concentration of fungal extracellular nucleic acids forms complexes with LL-37. The complexation probably modifies their stimulatory properties, leading to a change in the response of infiltrating neutrophils. This result may underscore the regulatory function of LL-37 in the immune system. NETosis, while is crucial for capturing and eliminating pathogens, can become problematic when it is overly activated. Excessive NETosis activation during infection leads to tissue damage, chronic inflammation, and autoimmune disorders, as it releases a broad spectrum of pro-inflammatory molecules (50). Therefore, the immune system may carefully balance the induction of NETosis to maximize pathogen clearance while minimizing collateral damage in tissue. In this context, LL-37 emerges as one of the key regulators of neutrophil response to fungal infections. By forming complexes with nucleic acids, LL-37 appears to regulate NETosis response, ensuring that it remains at a controlled and relatively low level.

*C. albicans* has evolved different strategies to evade the immune system. One of the mechanisms is proteolytic degradation of antimicrobial peptides. Our earlier research has revealed that the activity of different Saps leads to the enzymatic cleavage of the LL-37 peptide, generating shorter derivatives, mainly LL-25 (4). The resulting fragments have mostly impaired antimicrobial and immunomodulatory functions. Based on these findings, we extended our studies to evaluate the effects of these shorter LL-37 fragments on yeast nucleic acid complexation and their potential to induce NETosis. Remarkably, our data demonstrated a similar pattern to that observed with the native LL-37 peptide. This suggests that even in their truncated form, LL-37 fragments retain the capacity to interact with yeast nucleic acids and influence the activation of NETosis, despite their reduced antimicrobial effectiveness.

One of the primary mechanisms that enable neutrophils to ensure effective defense against fungal infection is the generation of ROS, which play a pivotal role in the neutrophil’s immune response, serving a dual function. First, they act as powerful antimicrobial agents, directly targeting and eliminating fungal pathogens. Secondly, ROS are also crucial signaling molecules that trigger NETosis and apoptosis (51, 52). Our experiments showed that the level of ROS generation during stimulation with DNA:LL-37 and RNA:LL-37 remains low, only slightly exceeding the levels observed in unstimulated cells. The insufficient level of ROS potentially explains the reduced NETosis in response to DNA:LL-37 and RNA:LL-37 stimulation. Further investigations have revealed that even a short pre-stimulation of neutrophils with DNA:LL-37 and RNA:LL-37 complexes results in the emergence of a neutrophils population that is resistant to the action of a strong activator of ROS production (PMA). The resulting population represented less than half of all neutrophils in our experiments, however, prolonging the pre-stimulation could make this effect more visible. It should also be noted that neutrophils are not a fully homogeneous group and some of them may be more susceptible to inhibitory effects. Similarly, we checked whether such pre-stimulation influences the induction of NETosis activated by PMA. The observed effect coincides with the results obtained for the ROS measurement. This finding strongly indicates a gradual activation of the inhibitory pathway downstream of PKC, which exerts a regulatory effect on NADPH oxidase activity. A similar observation, indicating the inhibition of ROS production and NETosis, in response to entire biofilm was presented by the group of Johnson et al. (28).

Neutrophil viability changes dynamically during infection. Many factors such as granulocyte-macrophage colony-stimulating factor (GM-CSF), IL-8, LPS and bacterial DNA containing unmethylated CpG motifs can prolong the longevity of neutrophils (42). Notably, LL-37, depending on its concentration, also modulate cell viability, inhibiting apoptosis at lower concentrations and promoting secondary necrosis at higher levels (53, 54). We examined the impact of DNA:LL-37 and RNA:LL-37 stimulation on neutrophil viability. Our analysis encompassed the examination of two key features of neutrophil apoptosis, the assessment of caspases activity and the level of phosphatidylserine residues exposure on the cell membrane (55). The evaluation of caspase 3/7 activity unveiled a significant anti-apoptotic impact of DN:LL-37 and RNA:LL-37 complexes. Interestingly, the observed caspase activity levels were even lower than in unstimulated cells, which naturally undergo spontaneous apoptosis upon *in vitro* culture. The examination of phosphatidylserine exposure levels demonstrated a similar trend. Stimulation using DNA:LL-37 and RNA:LL-37 complexes led to a notably reduced occurrence of early and late apoptotic features in the cell population. Importantly, this reduction was more pronounced compared to the stimulation with free LL-37, DNA, and RNA molecules. These findings are supported by the observed low levels of reactive oxygen species (ROS) accumulation, which are one of the apoptosis activators in neutrophils. To directly confirm anti-apoptotic activation we assessed the level of the anti-apoptotic Mcl-1 protein, which is the main regulator of neutrophil survival (42, 43). In all experiments, we observed higher Mcl-1 levels for stimulation with DNA:LL-37 and RNA:LL-37 than for the other stimulants, which confirms the activation of the anti-apoptotic pathway. This mechanism may have important implications in the context of inflammation, where increased neutrophil numbers and prolonged viability can contribute to a more robust immune defense.

Several previous studies in the field of autoimmunology have shown that the formation of self-DNA:LL-37 complexes may lead to an increased pro-inflammatory response. For example, the work of Zhang et al. has demonstrated that the complex of mitochondrial DNA with LL-37 exacerbates inflammation by stimulating IFNα production in plasmacytoid dendritic cells and inducing IL-6, IL-8, and TNFα release in neutrophils (40). The robust IL-8 release in neutrophils was also observed in the case of human RNA complexes with LL-37 in a psoriasis model (56). Therefore, in the final phase of the experiments, we assessed the effect of yeast nucleic acid complexation on the induction of neutrophil cytokine response. Our results indicated that DNA:LL-37 and RNA:LL-37 complexes predominantly enhance the production of IL-8. Notably, IL-8 serves as a pivotal chemotactic factor for neutrophils and other immune cells (57), and the observed increase in its levels may provide an explanation for the robust chemotaxis identified in response to DNA/RNA:LL-37 complexes.

In conclusion, our findings showed for the first time that the formation of *C. albicans* nucleic acid complexes with LL-37 may play a significant role in switching a neutrophil response, from rapid and uncontrolled NETosis, which can cause extensive destruction of local tissues, to a more prolonged mobilizing the whole immune system. Moreover, these results shed new light on observations made earlier regarding the blocking of NETs formation, upon neutrophil’s contact with a fungal biofilm. We also demonstrated the tremendous NETosis-blocking potential of the LL-37 peptide in contact with *C. albicans* nucleic acids, even as it is gradually degraded by the infecting pathogen exploiting the microbial proteolytic arsenal.

## Data availability statement

The datasets presented in this study can be found in online repositories. The names of the repository/repositories and accession number(s) can be found below: The data for publication “Complexation of fungal extracellular nucleic acids by host LL-37 peptide shapes neutrophil response to Candida albicans biofilm” can be found in RODBUK Cracow Open Research Data Repository: https://doi.org/10.57903/UJ/OAIR0H.

## Ethics statement

Ethical approval was not required for the human studies as blood samples were commercially purchased from the Regional Blood Center in Krakow, Poland. For human subject confidentiality assurances, blood material is deidentified thus this project adheres to appropriate exclusions from human subject approval. The team had no contact with donors and conducted no medical procedures on patients, making bioethics committee approval unnecessary. The studies complied with the national legislation and institutional requirements. Written informed consent for participation was not required from the participants or the participants’ legal guardians/next of kin due to the nature of working with commercially acquired blood products.

## Author contributions

MJ: Conceptualization, Data curation, Formal analysis, Investigation, Methodology, Writing – original draft, Writing – review & editing. MZ: Conceptualization, Investigation, Methodology, Supervision, Writing – review & editing. MR-K: Conceptualization, Formal analysis, Funding acquisition, Methodology, Project administration, Supervision, Writing – review & editing.
