# Supplementary material for: Complexation of fungal extracellular nucleic acids by host LL-37 peptide shapes neutrophil response to Candida albicans biofilm
Source: Front Immunol. 2024 Feb 6;15:1295168. doi: 10.3389/fimmu.2024.1295168 (PMC10880380; doi:10.3389/fimmu.2024.1295168)
Supplement: Supplementary file 1 [file DataSheet_1.pdf]

Supplementary Material

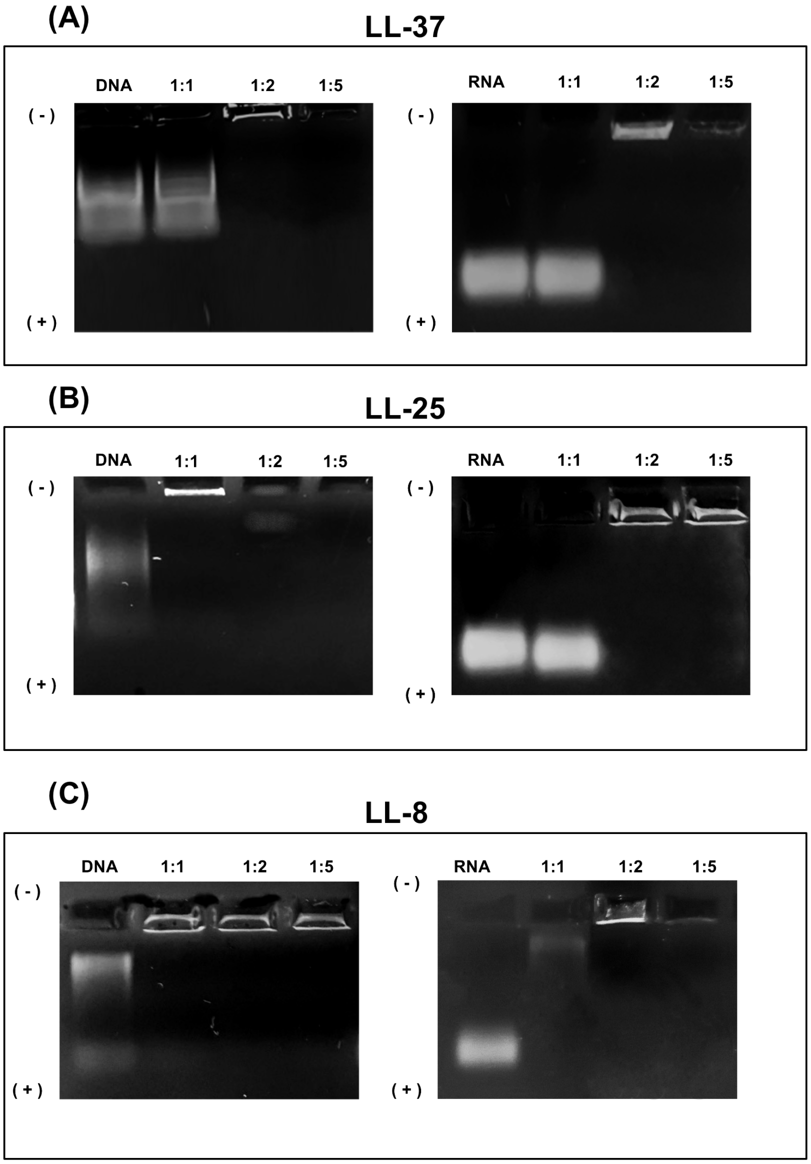

Supplementary Figure 1. Optimalization of *C. albicans* nucleic acid complexes formed with LL-37, LL-25, LL-8. The isolated DNA and RNA (10 µg) were incubated with LL-37, LL-25, LL-8 in mass ratios 1:1, 1:2, 1:5 for 30 minutes at 37°C in PBS. Then, 2 µl of Midori Green Advance DNA Stain was added to each probe. The samples (10 µg) were separated on a 1% agarose gel (80V for 15 minutes). The electrophoretic image was obtained using a BioRad Imaging Systems.
